# Dietary diversity and poverty as risk factors for leprosy in Indonesia: A case-control study

**DOI:** 10.1371/journal.pntd.0006317

**Published:** 2018-03-13

**Authors:** Salma Oktaria, Norma Sofisa Hurif, Wardiansyah Naim, Hok Bing Thio, Tamar E. C. Nijsten, Jan Hendrik Richardus

**Affiliations:** 1 Department of Dermatology, Erasmus University Medical Center, Rotterdam, the Netherlands; 2 Department of Dermatology and Venerology, Faculty of Medicine Universitas Indonesia, Jakarta, Indonesia; 3 Department of Public Health, Erasmus University Medical Center, Rotterdam, the Netherlands; 4 Department of Epidemiology, Faculty of Public Health, Airlangga University, Surabaya, Indonesia; University of California San Diego School of Medicine, UNITED STATES

## Abstract

**Background:**

Poverty has long been considered a risk factor for leprosy and is related to nutritional deficiencies. In this study, we aim to investigate the association between poverty-related diet and nutrition with leprosy.

**Methodology/Principal findings:**

In rural leprosy-endemic areas in Indonesia, we conducted a household-based case-control study using two controls for each case patient (100 recently diagnosed leprosy patients and 200 controls), matched for age and gender. All participants were interviewed to collect information on their demographics, socioeconomic situation, health, and diet. Body mass index, dietary diversity score, as well as anemia and iron micronutrient profiles were also obtained. By means of univariate, block-wise multivariate, and integrated logistic regression analyses, we calculated odds ratios between the variables and the occurrence of leprosy. Unstable income (odds ratio [OR], 5.67; 95% confidence interval [CI], 2.54–12.64; p = 0.000), anemia (OR, 4.01; 95% CI, 2.10–7.64; p = 0.000), and higher household food insecurity (OR, 1.13; 95% CI, 1.06–1.21; p = 0.000) are significantly associated with an increased risk of having leprosy. Meanwhile, higher education (OR, 0.34; 95% CI, 0.15–0.77; p = 0.009) and land ownership (OR, 0.39; 95% CI, 0.18–0.86; p = 0.019) have significant protective associations against leprosy. Although lower dietary diversity, lack of food stock, food shortage, low serum iron, and high ferritin were found more commonly in those with leprosy, the occurrence of leprosy was not significantly associated with iron deficiency (OR, 1.06; 95% CI, 0.10–11.37; p = 0.963).

**Conclusions/Significance:**

Food poverty is an important risk factor for leprosy susceptibility, yet the mechanisms underlying this association other than nutrient deficiencies still need to be identified. With a stable incidence rate of leprosy despite the implementation of chemoprophylaxis and multidrug therapy, improving dietary diversity through food-based approaches should be initiated and directed toward high-prevalence villages. The possible underlying factors that link poverty to leprosy other than nutrient deficiencies also need to be identified.

## Author summary

Despite the suggestion that nutritional deficiencies may impair host immune responses against *Mycobacterium leprae*, there has not been any systematic study on how various aspects of poverty interact and associate with nutrition and leprosy. In poor rural areas in Indonesia that have the highest proportion of multibacillary cases, we aimed to investigate these associations by interviewing recently leprosy diagnosed patients and measuring their anemia and iron profiles. Our findings suggested that, compared to the control population, people who are at an increased risk of contracting leprosy have lower education, lack of stable income to provide diverse types of food, and are anemic. Although low serum iron and high ferritin levels were found more commonly in those with leprosy, we did not find a significant association between iron deficiency and leprosy. Our study clarifies that food poverty is an important risk factor for leprosy susceptibility, yet the mechanisms underlying the association between diet and leprosy other than nutrient deficiencies still need to be identified. Improving dietary diversity through food-based approaches should be initiated and directed towards high-prevalence villages.

## Introduction

Leprosy has long been known as a disease of poverty, yet the mechanism underlying this interaction remains unclear. Most of the affected countries are underdeveloped, in which people affected by leprosy are born and raised in poor environments and continue being pushed into poverty due to the stigma and disabilities [1]. Poverty means more than just a lack of income; it also encompasses the multiplicity of non-monetary aspects that often combine and intensify the negative effects of being poor, including lack of proper food and nutrient intakes [2]. Correspondingly, food shortage, food insecurity, and lower dietary diversity are several aspects of poverty that are more commonly found in those struggling with leprosy [3]. Previous studies have shown positive associations between food shortage and food insecurity with the occurrence of leprosy, and it was suggested that impaired host immune response against the causative bacteria as a result of insufficient nutritional intake is the possible cause of this condition [4]. However, there has been no systematic study on how various aspects of poverty interact and associate with leprosy to support the suggestion, particularly in Indonesia, which is currently the home of more than 17,000 new leprosy cases registered annually and has the highest proportion of multibacillary (MB) cases [5]. The purpose of this research, which is a part of the MicroLep Study, is to elucidate the association between poverty-related dietary intake and leprosy by determining the interaction between demographic, socioeconomic, and diet-related factors of poverty on several nutrition indicators, which encompasses people with leprosy and healthy controls in the Indonesian population.

## Methods

### Ethics statement

This study was approved by the ethical review committee of the Faculty of Medicine, Universitas Indonesia, Jakarta, Indonesia (reference number: 595/UN2.F1/ETIK/2016). The Agency for National and Political Unity of Bangkalan and the District Health Office of Bangkalan, Madura, East Java Province, Indonesia have also been informed about this study and have given their approval and support prior to the beginning of the study. A signed informed consent form was obtained from each participant before starting the study.

### Study design and population

We conducted a household-based, case-control study in rural areas of Bangkalan, Madura, East Java, Indonesia, from November to December 2016. Bangkalan has 22.38% inhabitants who are living below the poverty line, making it the second poorest region in Madura after its neighboring district, Sampang [6]. Correspondingly, this area is also endemic for leprosy; 310 new cases were diagnosed in 2015 in a total population of 1 million, yet no chemoprophylaxis therapy has been given to prevent leprosy in patient contacts [7,8].

Data on people with leprosy were gathered from the Leprosy Cohort Data Report of the Bangkalan Municipality of Health. Seventeen of 22 primary health care facilities in Bangkalan participated in this study. Selected cases between the ages of 18 and 65 who were being actively treated with the World Health Organization (WHO)-recommended multidrug therapy (MDT) regimen were chosen based on the current cohort report up to September 2016. Control subjects who lived in the village or neighborhood with common characteristics as the cases with the same sex and age range were also selected, with a ratio of 1:2 between the cases and controls. The following exclusion criteria were applied: refusal to participate, limited understanding of information, pregnant or breastfeeding, or had taken systemic antibiotics other than the WHO-MDT regimen within 30 days preceding inclusion. Additionally, control subjects who had household members with a history or newly diagnosed leprosy at the time of inclusion were also excluded.

### Data collection

Six trained surveyors and six trained health workers who spoke Bahasa Indonesia and Madurese collected the data using a structured questionnaire along with peripheral blood sample collection during household visits.

The questionnaire used in this research was adapted from a study that was conducted in Bangladesh [9]. The original English version of the questionnaire had been translated into Bahasa Indonesia, optimized, pre-tested, and validated prior to the study. The first section of the questionnaire focused on the demographic, socioeconomic, and health characteristics of the subjects and their households. Household size (the number of people eating in the house), occupation of the income generator and subject, land ownership, as well as the subject’s level of education, average income, income variation, food expense, and self-classification on a poverty scale were registered. The triggering cause of income variation was also included, but the difference between pre- and post-leprosy diagnosis was not asked due to the tremendous stigma in Bangkalan. As for the health characteristics, a number of questions were asked about the details of any acute and chronic diseases in the prior year, the presence of a BCG scar, history of medication, and leprosy diagnosis (for the case group). Afterward, the household food insecurity access scale (HFIAS) was administered to specify the problem concerning food insecurity during the preceding four weeks [10]. Food storage and dietary modification such as lessening the number or variety of meals was also asked in detail. For comparability purposes, food shortage was defined with the same criteria as in the Bangladesh study [1]. Finally, dietary intakes consisting of three meals a day and snacks in between were assessed by 24-hour recall, from which the individual dietary diversity score (IDDS) was calculated. The subjects described their 24-hour food consumption history in chronological order, starting from breakfast the previous day. The details of ingredients for each meal and snack were obtained, particularly for mixed dishes and processed foods. However, the food quantity was not obtained as the 24-hour recall focused on the quality of the diet composition. Subsequently, the food ingredients were categorized into nine categories and were calculated based on the Food and Nutrition Technical Assistance Project/Food and Agriculture Organization of the United Nations (FANTA/FAO) guidelines [11]. “Milk and milk products” were defined as all dairy-based products with the exception of butter, and the slight amount of milk in coffee was not counted. Moreover, garlics, shallots, and chili spices were classified as condiments due to the small amounts consumed. Considering that special feasts are usually prepared for special celebrations or specific religious holidays in Indonesia, 24-hour recall was not carried out during those particular days. Thus, we could assume that the variance among food ingredients remained stable over the period.

Following the interview, weight was assessed using a portable scale (GEA Medical, Jakarta, Indonesia) and height was measured using a measuring tape; the subjects were asked to remove their footwear and stand on a flat surface with their back against the wall. Peripheral blood from both groups was collected into EDTA and SST vacutainers (BD, Franklin Lakes, NJ, USA) by trained health workers and distributed to a laboratory in Surabaya, where blood tests were performed to measure hemoglobin and iron micronutrient profiles.

### Data management and statistical analyses

**Fig 1:**
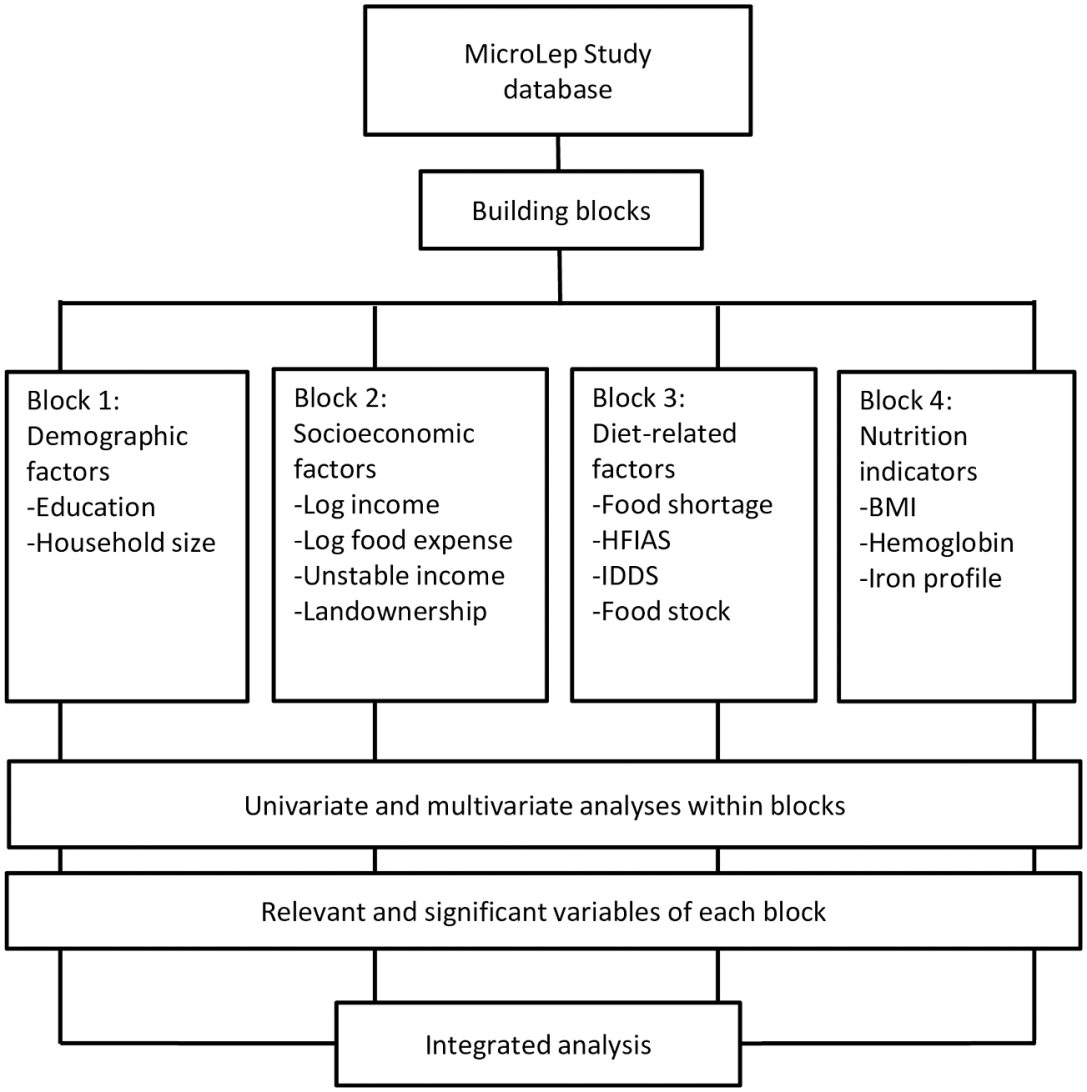
Data analyses flowchart.

A MicroLep Study database, designed in Microsoft Excel, was established and the data were entered by well-trained data-entry personnel. Demographic, socioeconomic, and health characteristics were determined with descriptive analyses. Subsequently, four blocks consisting of several related variables were built into a framework (Fig 1). Univariate, block-wise multivariate, and integrated analyses were performed using logistic regression in SPSS version 21 with case or control as the dependent variable. Sex and age were also adjusted in order to control for confounding effects from the pair matching design [12]. Univariate and multivariate analyses within the blocks were performed first, and the variables that were relevant and significantly associated with leprosy from each block (p<0.05) were included in the integrated analysis.

## Results

### Subjects’ characteristics

A total of 276 of 419 cases were eligible for the study, of which 103 patients were randomly selected using Research Randomizer. However, only 100 cases were included in the analysis due to uncomplete data. Among 218 house visits, 200 controls were able to complete the questionnaire and were included in this study (response rate: 91.74%). In total, 300 subjects consisting of 100 cases and 200 controls were included in the analysis, with a mean age of around 35 to 36 years and approximately equal numbers of males and females. The majority of cases were MB (89%), of which 15% and 6% of patients presented with grade 1 and 2 disabilities, respectively. The demographic, socioeconomic, and health characteristic of the subjects are shown in Table 1.

**Table 1:** Demographic, socioeconomic, and health characteristics.

Characteristic	Cases (N = 100)	Controls (N = 200)
Sex, male	52.0%	52.0%
Age		
Mean, y	39.75±14.00	39.74±13.85
16–29	30.0%	30.5%
30–44	27.0%	28.5%
45–65	43.0%	41.0%
Household size, mean	5.05±2.10	5.46±2.59
Education		
No education	52.0%	31.5%
Primary education	25.0%	31.0%
High education	23.0%	37.5%
Occupation		
Unemployed	27.0%	28.5%
Farmer	46.0%	47.0%
Labor	14.0%	9.5%
Employed	13.0%	15.0%
Income, IDR, mean	997,389±82,721	1,063,182±64,0837
Food expenditure, IDR, mean	694,564±36,002	827,850±419,612
Land ownership, landowner	54.0%	61.0%
Self-classification		
Very Poor	3.0%	0.0%
Poor	22.0%	16.5%
Low-middle income	47.0%	51.0%
Middle income	26.0%	32.0%
Rich	2.0%	0.5%
History of disease past year		
Acute disease	1.7%	5.0%
Chronic disease	15.0%	11.5%
BCG, vaccinated	36%	40.5%
Type of leprosy		
Paucibacillary (PB)	11.0%	0.0%
Multibacillary (MB)	89.0%	0.0%
Disability, yes	21.0%	0.0%

### Diet-related factors

**Fig 2:**
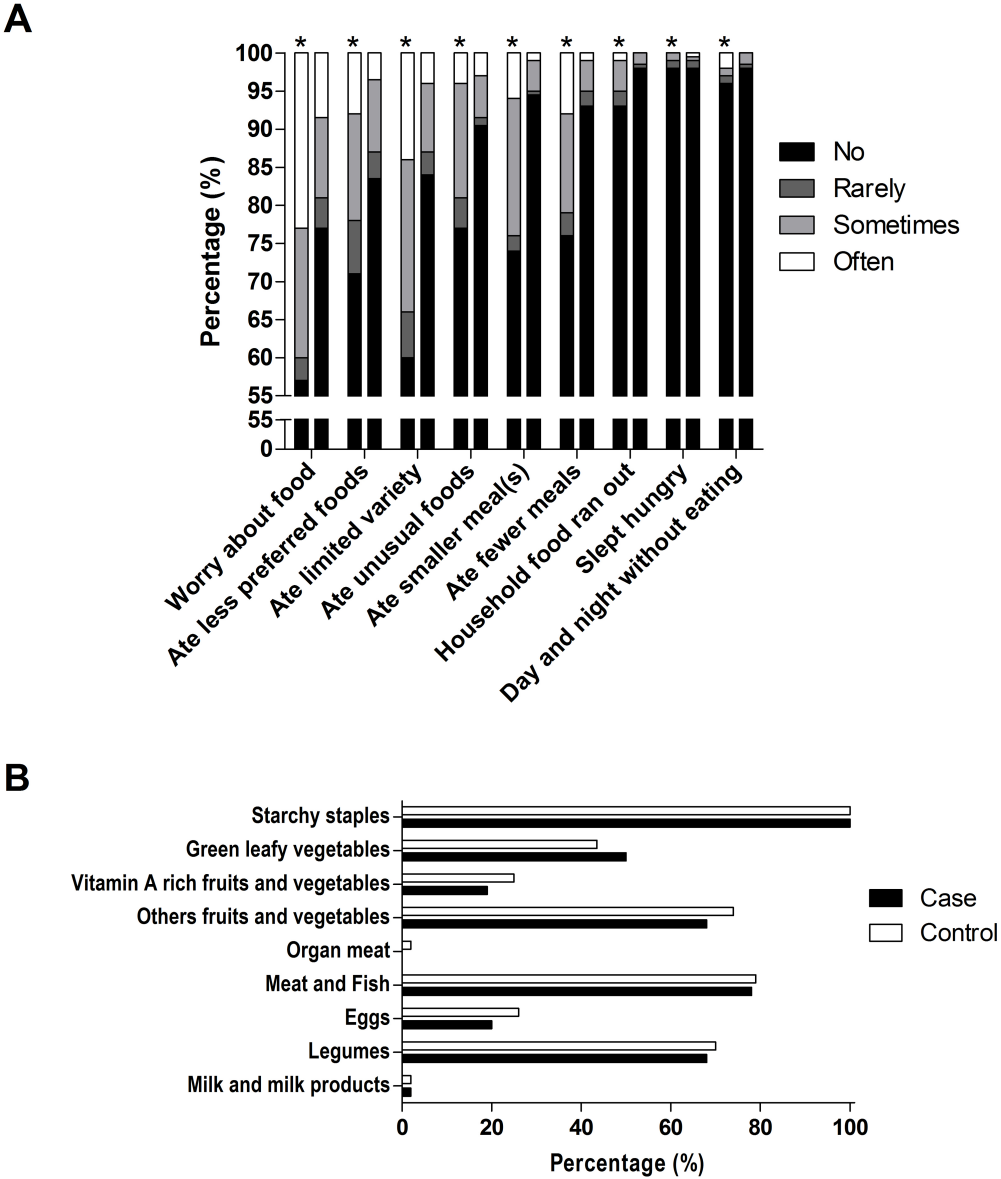
Comparison of the frequency-of-occurrence of each HFIAS item (A) and the IDDS profile (B) between the cases and controls.

Detailed information about HFIAS, food shortage, and IDDS are provided in Table 2 and Fig 2.

**Table 2:** Food insecurity, food shortage, diet modifications, food storage, and dietary diversity.

		Cases (N = 100)	Controls (N = 200)
HFIAS score, mean		4.33±5.20	1.73±3.50
HFIAS category			
Food secure		45.0%	72.0%
Mildly food insecure		8.0%	11.0%
Moderately food insecure		30.0%	12.5%
Severely food insecure		17.0%	4.5%
Experienced food shortage at any time in life		57.0%	35.0%
Food shortage repeated frequently		65.9%	50.0%
Diet modification			
Reduce frequency meals		29.5%	29.3%
Reduce food variance		43.2%	43.9%
Reduce both frequency and variance		27.4%	26.8%
Changes in food variances consumption			
Rice	No change	44.9%	71.8%
	Reduce	36.9%	28.2%
	Give up	18.2%	0.0%
Vegetables	No change	93.8%	77.6%
	Reduce	6.2%	7.0%
	Give up	0.0%	15.4%
Meat	No change	63.6%	44.6%
	Reduce	0.0%	16.9%
	Give up	36.4%	38.5%
Fish	No change	2.2%	40.3%
	Reduce	52.3%	36.6%
	Give up	45.5%	23.1%
Legumes	No change	98.5%	97.2%
	Reduce	1.5%	2.8%
	Give up	0.0%	0.0%
Fruits	No change	96.9%	68.4%
	Reduce	3.1%	8.5%
	Give up	0.0%	23.1%
Household food storage, yes		55.0%	68.0%
Duration food-storage, weeks, mean		4.90±11.53	6.49±11.91
IDDS		3.71±1.10	4.06±1.17

Note: HFIAS, household food insecurity access scale; IDDS, individual dietary diversity score.

The HFIAS score was two times higher in people with leprosy compared to the controls (p = 0.000). In addition, food storage availability was 18% higher in the controls, which lasted on average of 6.5 weeks compared to 5 weeks in people with leprosy.

### Nutrition indicators

A paired t-test showed that the mean of the BMI between the cases and controls was significantly different (p = 0.002). Moreover, around 42% of the people with leprosy had anemia [13], which was 29% higher than the controls. Reflecting micronutrient deficiency, the blood iron profile showed that more people with leprosy had low iron serum levels than the controls (23% and 9%, respectively). Total iron-binding capacity (TIBC) and transferrin saturation were also lower (Table 3), while high ferritin levels were twice as common in those with leprosy than in the controls (37% and 16%, respectively).

**Table 3:** Body mass index, anemia and iron profiles.

	Cases (n = 100)	Controls (N = 200)
BMI		
Mean, kg/m^2^	22.09±4.91	22.89±4.43
Underweight	22.0%	12.5%
Normal weight	53.0%	65.0%
Overweight	20.0%	13.0%
Obese	5.0%	9.5%
Hemoglobin		
No anemia	58 (58.0%)	173 (86.5%)
Anemia	42 (42.0%)	27 (13.5%)
Iron		
Under	23 (23.0%)	18 (9.0%)
Normal	77 (77.0%)	182 (89.5%)
TIBC		
Under	16 (16.0%)	12 (6.0%)
Normal	84 (84.0%)	188 (94.0%)
Ferritin		
Under	6 (6.0%)	8 (4.0%)
Normal	57 (57.0%)	160 (80.0%)
Higher	37 (37.0%)	32 (16.0%)
Transferrin saturation		
Under	16 (16.0%)	20 (10.0%)
Normal	84 (84.0%)	180 (90.0%)

Note: BMI, body mass index (underweight <18.5 kg/m2; normal 18.5–25 kg/m2; overweight 25–30 kg/m2; obese >30 kg/m2); normal laboratory values: hemoglobin (male ≥13 g/dL, female ≥12 g/dL); iron (male 59–158 μg/dL, female 37–145 μg/dL); TIBC, total iron binding capacity (228–428 μg/d); ferritin (male 15–200 ng/dL, female 15–150 ng/dL); transferrin saturation 16–60%.

### Block-wise and integrated analyses

The results of univariate and multivariate analyses per block are shown in Table 4. First, education dominated the demographic factors block (p<0.05); higher education was associated with a lower risk of leprosy.

**Table 4:** Block-wise univariate and multivariate logistic regression analyses.

	Cases N = 100	Controls N = 200	Univariate* OR (95% CI)	Block-wise multivariate* OR (95% CI)
**Block 1: Demographic factors**				
Education				
No education	52 (52.0%)	63 (31.5%)	1.0**	1.0**
Primary	25 (25.0%)	62 (31.0%)	0.49(0.27–0.88)**	0.39 (0.20–0.74)**
High education	23 (23.0%)	75 (37.5%)	0.37 (0.20–0.67)**	0.24 (0.12–0.50)**
Household size, mean	5.05±2.10	5.47 ±2.59	0.93 (0.84–1.03)	0.92 (0.82–1.02)
**Block 2: Socioeconomic factors**				
Income per capita (log), mean	5.18±0.43	5.25±0.31	0.57 (0.29–1.12)	0.46 (0.16–1.34)
Unstable income				
No	37 (37.0%)	122 (61.0%)		
Yes	63 (63.0%)	78 (39.0%)	2.69 (1.63–4.42)**	5.02(2.39–10.50)**
Food expense per capita (log), mean	5.13±0.33	5.17±0.26	0.58 (0.25–1.34)	1.31 (0.36–4.83)
Land ownership				
No	54 (54.0%)	122 (61.0%)		
Yes	46 (46.0%)	78 (39.0%)	1.33 (0.82–2.16)	0.40 (0.19–0.84)**
**Block 3: Diet-related factors**				
Food shortage in life				
No	43 (43.0%)	130 (65.0%)		
Yes	57 (57.0%)	70 (35.0%)	2.49 (1.51–4.08)**	1.63 (0.92–2.90)
Food stock availability				
No	45 (45.0%)	64 (32.0%)		
Yes	55 (55.0%)	136 (68.0%)	0.58 (0.35–0.94)**	0.61 (0.36–1.04)
HFIAS, 0–27	4.33±5.20	1.73±3.5	1.15 (1.08–1.21)**	1.11 (1.02–1.20)**
IDDS, 0–9	3.71±1.10	4.06±1.17	0.76 (0.62–0.95)**	0.81 (0.64–1.02)
**Block 4: Nutrition indicators**				
BMI (kg/m^2^)	22.09±4.91	22.89±4.43	0.96 (0.91–1.01)	0.96 (0.90–1.02)
Hemoglobin				
No anemia	58 (58.0%)	173 (86.5%)		
Anemia	42 (42.0%)	27 (13.5%)	4.85 (2.72–8.67)**	4.90 (2.50–9.61)**
Iron				
Normal	76 (76.0%)	179 (89.5%)		
Under(U)	24 (24.0%)	21 (10.5%)	2.72 (1.42–5.19)**	1.06 (0.10–11.37)
TIBC				
Normal	36 (36.0%)	90 (45.0%)		
Under(U)	64 (64.0%)	110 (55.0%)	1.52 (0.90–2.56)	0.99 (0.53–1.85)
Ferritin				
Under(U)	6 (6.0%)	8 (4.0%)	1.0**	1.0**
Normal	57 (57.0%)	160 (80.0%)	0.491 (0.16–1.49)	1.88 (0.23–15.34)
Higher	37 (37.0%)	32 (16.0%)	1.64 (0.50–5.35)	7.70 (0.84–70.81)
Saturation transferrin				
Normal	84 (84.0%)	180 (90.0%)		
Under(U)	16 (16.0%)	20 (10.0%)	1.73 (0.85–3.54)	1.65 (0.49–5.56)
Iron (U)*TIBC(U)			4.97 (2.10–11.75)**	7.17 (0.84–61.06)
Ferritin*Iron			1.0**	1.0
Ferritin(H)*Iron(U)			5.95 (1.79–19.70)**	0.20 (0.11–24.72)
Ferritin(N)*Iron(U)			1.96 (0.81–4.78)	0.39 (0.33–44.50)

Note:

*, adjusted for age and sex;

**, p<0.05;

HFIAS, household food insecurity access scale; IDDS, individual dietary diversity score; BMI, body mass index; TIBC, total iron binding capacity.

Second, unstable income and land ownership played an important role (p<0.05) in the socioeconomic factors block; people with these factors had a greater risk of developing leprosy. In addition, both log income per capita and log food expense had a protective association against leprosy, yet the numbers were almost similar in both groups and therefore did not show significant associations with leprosy.

Third, all variables in the diet-related factors block were significantly associated with leprosy in the univariate analysis. High HFIAS and experiencing food shortage at any time in life increased the risk of having leprosy, while IDDS and food stock availability had a reverse association with leprosy. Nevertheless, only HFIAS remained significant in the multivariate analysis (p<0.05).

Fourth, all variables in the nutrition indicators block other than BMI also showed significant associations with leprosy in the univariate analysis. In this block, in addition to analyzing the original variables, we also considered the interactions between iron-TIBC and iron-ferritin [14–16], which were also statistically significant in the univariate analysis. However, only hemoglobin remained significant (p<0.05) in the multivariate analysis.

Following per block multivariate analyses, all of the significant and relevant variables were included in an integrated analysis. This final analysis aimed to reveal the connection among variables from each block. The results are presented in Table 5. Based on this analysis, variables of education, unstable income, HFIAS, and hemoglobin remained significantly associated with leprosy (p<0.05).

**Table 5:** Integrated logistic regression analysis consisting of significant and relevant variables.

Factors	Integrated analysis		
	OR*	(95% CI)	p-value
Education			
No Education	1.00		0.008**
Primary Education	0.36	(0.18–0.75)	0.006**
High Education	0.34	(0.15–0.77)	0.009**
Unstable income			
No	1.00		
Yes	5.67	(2.54–12.64)	0.000**
Land ownership			
No	1.00		
Yes	0.39	(0.18–0.86)	0.019**
HFIAS, mean	1.13	(1.06–1.21)	0.000**
IDDS, mean	0.85	(0.67–1.09)	0.213
Hemoglobin			
Non anemia	1.00		
Anemia	4.01	(2.10–7.64)	0.000**

Note:

*, OR adjusted for age, sex, and all variables in the column;

**, p<0.05;

R^2^: 0.413; HFIAS, household food insecurity access scale; IDDS, individual dietary diversity score.

## Discussion

Our results showed that people with leprosy have less favorable socioeconomic and demographic conditions, as well as dietary consumption. Low education levels, unstable incomes, and no land ownership are some aspects of poverty that were associated with the risk of having leprosy. Moreover, although the iron profiles were not significantly associated with leprosy, the low nutritional status in people with leprosy was associated with lower IDDS and higher HFIAS.

### Education level

Based on our analysis, education level had a protective association against leprosy. Thus, the more educated someone is, the lower chance they will contract leprosy. In this sense, education is regarded as a substantial factor of subjects’ self-awareness that contributes to disease elimination. This is consistent with a previous study in Brazil, where the patients were unlikely to report their symptoms to receive treatment or did not even know that they had leprosy due to lack of knowledge and awareness of the disease [17]. Additionally, a higher education level is also often associated with better economic outcomes.

### Unstable income and land ownership

Other important factors associated with leprosy were unstable income and land ownership, which are related to income inequality. Based on our analysis, people with unstable incomes were five times more likely to develop leprosy, while owning private land decreased the risk of getting leprosy by 60% (OR = 0.39 [CI 0.18–0.86], p = 0.019). While those with assets are able to provide better and more stable socioeconomic outcomes [1], freelance workers such as farmers and labors have only seasonal incomes from seasonal jobs [18,19].

### Food expenditures and diet-related factors

In contrast to the Bangladesh study [9], our research did not find a difference in food expenditures between those with leprosy and the controls. Limited food preference, culture, and food availability in the study areas might have contributed to this value. However, heterogeneities in food consumption may vary across households even with the same food expenditures and can still influence the subjects’ nutritional intake [20]. This is consistent with our result on IDDS, which was significantly lower in the case group, who ate fewer fruits and vegetables, eggs, and legumes, nuts, and seed products (Fig 2b). Although it was not statistically significant, IDDS had a reverse association with leprosy (OR = 0.85 [CI 0.67–1.09], p = 0.213). In contrast, a higher HFIAS score was significantly associated with a higher chance of contracting leprosy (OR = 1.13 [CI 1.06–1.21], p = 0.000). Nevertheless, our score was lower than in the Bangladesh study [9]. Lower gross domestic product (GDP) per capita at purchasing power parity (PPP) in Bangladesh ($3,581) than in Indonesia ($11,612) may explain these findings [21].

In terms of food shortage, our study showed similar results with those of Feenstra *et al* [1] (Table 4). In the univariate analysis, around 53% of cases also experienced food shortage at some time in their lives (with a mean length of 42.84±70.49 weeks) that was significantly associated with leprosy. Although this was not statistically significant in the integrated analysis, in theory, a prolonged food shortage may result in a deficiency of essential nutrients that are needed to boost an adequate immune response against infectious agents [22], thus increasing the risk of contracting infectious diseases.

### Nutrition and leprosy

Based on our integrated analysis, those with anemia are at an increased risk of contracting leprosy (OR = 4.01 [CI 2.10–7.64], p = 0.000). There are several underlying conditions related to anemia, such as micronutrient deficiencies [23,24], infectious diseases [25], and hereditary conditions (thalassemia) [16]. Iron deficiency characterized by high TIBC or low ferritin is the most common cause of anemia. However, diagnosing iron deficiency anemia (IDA) in particular areas where infectious diseases are prevalent can be very challenging as serum ferritin levels may increase due to immune responses to the infectious agent, masking a pure iron deficiency diagnosis [16]. The iron profiles in our study population were consistent with anemia that is caused either by chronic diseases (ACD) or mixed IDA and ACD. Dietary intake has been reported to influence either hemoglobin or iron levels [26], and from the interview, we knew that those with leprosy consumed much less red meat and eggs, which are rich in iron. Hence, iron deficiency from a less diverse diet mixed with chronic infection by *M*. *leprae* might be the cause of anemia in this study. Our additional multivariable analyses demonstrated that lower dietary diversity and higher HFIAS scores escalated the risk of anemia (OR = 0.86 [CI 0.67–1.10], p = 0.227) and OR = 1.09 [CI 1.03–1.16], p = 0.003, respectively) and that dietary diversity had a reverse association with TIBC levels, which is a sensitive indicator of iron deficiency (OR = 1.37 [CI 0.60–3.11], p = 0.454). However, our final findings do not support the suggestion that iron micronutrient deficiency due to insufficient nutritional intake increases the susceptibility to leprosy. More studies are needed to identify other possible mechanisms underlying the association between poverty-related diet and leprosy. For instance, if diet-related risk factors for leprosy result from altering the skin or gut microbiota composition. Further research is currently being conducted to elucidate the role of diet-microbiota interaction in leprosy.

Although this study was carefully prepared and conducted, there were some unavoidable limitations. First, the data were collected after the diagnosis of leprosy and due to the strong stigma in the research areas, we were not allowed to specifically ask for any changes in the subjects’ income and diet after diagnosis, which made it hard to determine a causal relationship. Furthermore, the data regarding the subjects’ dietary intake were collected using a cross-sectional design. Ideally, a longitudinal study on diet and health should be conducted to compare data between those who eventually develop leprosy and those who do not. However, leprosy is a slowly developing infectious disease with a very long incubation period, so it is still difficult to determine a causal relationship using a short-term longitudinal study. In order to correct this difference, we asked all subjects for any changes in their economic status and dietary intake in general. All of the subjects had anonymously answered that they had been experiencing the same conditions and mostly consuming the same diet in the prior years. Only two subjects indicated a variation in their income due to their health, but not specifically for leprosy.

Second, the subjects were asked to reveal their dietary intake and food shortage history in the past 24 hours, past year, and in longer periods, introducing recall and response biases. In order to limit the effects on our results, the same questions were asked several times and atypical days such as parties or religious holidays were avoided. Third, in order to assess dietary intake more objectively, biomarkers for other micro- and macronutrients in blood should also be analyzed. However, we analyzed only the iron profiles considering the cost, positive correlations with dietary intake, their essential role during infection, and the limited research on the role of this micronutrient in leprosy.

In conclusion, our findings suggest that food poverty is an important risk factor for leprosy susceptibility, yet the mechanisms underlying this association other than nutrient deficiencies still need to be identified. With a relatively stable incidence rate of leprosy despite the implementation of chemoprophylaxis and multidrug therapy, improving dietary diversity through food-based approaches should be initiated and directed toward high-prevalence villages. The possible underlying factors that link poverty to leprosy other than nutrient deficiencies also need to be identified.

## Supporting information

S1 Checklist STROBE checklist. (DOCX)
